# Cure of Vesico-Vaginal Fistula, in a Novel Way

**Published:** 1857-07

**Authors:** 

**Affiliations:** L’Union Médicale


*Cure of Vesico-Vaginal Fistula in a Novel Way.* By Dr. BERTEL. (L'Union Médicale.) Dr. Bertel records a case of cure of vesico-vaginal fistula by a method which consists in pinching and crushing the vaginal mucous membrane. A woman, aged fifty, had suffered from a fistula for fourteen years. It was deeply seated, and engaged the body of the bladder on a level with the os tincæ. It was capable of admitting the tip of the finger through the vagina into the bladder. It was slightly oval; its larger extremity was directed towards the fundus of the bladder; its edges were somewhat thickened and hard; it was not funnel-shaped. The lesion followed a laborious delivery. M Bertel applied a pinching instrument to nip the edges of the fistula together, which he promises to describe hereafter when made more presentable and scientific. On the third day it was found that no urine escaped into the vagina. On removing the instrument, the opening was found closed. In its place was a ridge of a reddish brown color, easily bleeding, half the size of a cherry. Henceforth all urine passed by the urethra—no opening could be detected. The cure Dr. Bertel describes as perfect.—*Virginia Medical Jour.*

